# Vitrectomy using 0.025% povidone-iodine irrigation for treating post-traumatic endophthalmitis due to intraocular foreign bodies: Two case reports

**DOI:** 10.3389/fsurg.2022.988776

**Published:** 2023-01-20

**Authors:** Chi Liu, Ke Xu, Ying Hu, Xiaotong Zhuang, Bo Fu, Lin Wang, Xinzhu Jia, Li Xu

**Affiliations:** Department of Ophthalmology, Shenyang Fourth People's Hospital, Shenyang, China

**Keywords:** 0.025% povidone-iodine, balanced salt solution PLUS, traumatic endophthalmitis, case report, foreign body (F.B)

## Abstract

Traumatic eye injury-related endophthalmitis is a serious traumatic complication that threatens the vision of many patients worldwide. Herein, we present two cases of traumatic endophthalmitis that underwent 0.025% povidone-iodine treatment and hoped to introduce the bactericidal effect of 0.025% povidone-iodine in balanced salt solution PLUS (0.025% PI-BSS PLUS) and its use in vitrectomy for traumatic endophthalmitis. The 0.025% PI-BSS PLUS solution is bactericidal and nontoxic when used as an irrigation solution in pars plana vitrectomy. The two cases of traumatic endophthalmitis were resolved by pars plana vitrectomy using 0.025% PI-BSS PLUS.

## Introduction

Traumatic eye injury is one of the most devastating attacks on visual acuity, particularly attributed to intraocular foreign bodies (1). Initial treatments for intraocular foreign bodies include suturing and taking out the foreign bodies, in which preventing post-traumatic endophthalmitis by controlling bacterial growth is an essential process. In terms of the initial treatment for endophthalmitis, intravitreal injection (IVI) of antibiotics, such as vancomycin or ceftazidime, has been widely recommended in clinical practice (2). However, multidrug-resistant organisms resistant to both of these antibiotics have already been reported (3). Compared with antibiotics, povidone-iodine (PI) exhibits a wide range of microbicidal actions against multidrug-resistant bacteria (4), candida (5), viruses (6), and acanthamoeba (7) and is also active against biofilms (8–11). We report two cases diagnosed as traumatic endophthalmitis with intraocular foreign bodies, treated with IVI of 0.1 ml/1.25% PI, followed by vitrectomy using a 0.025% PI irrigation solution during the surgery. The current mixture was prepared to achieve a uniform concentration. We further reduced the volume to 0.1 ml (i.e., to 0.1 ml of 1.25% PI), which was used for IVI with a 25G needle. Assuming that the vitreous volume is 5 ml, the vitreous concentration of PI was calculated to be 0.025%, the same as the 0.025% PI-BSS PLUS used as an irrigation solution for vitrectomy in eyes with endophthalmitis (12). The present study was approved by the Ethics Committee of Shenyang Fourth People's Hospital. All procedures conformed to the Declaration of Helsinki, and informed consent was obtained from two patients.

## Case 1

**Figure 1:**
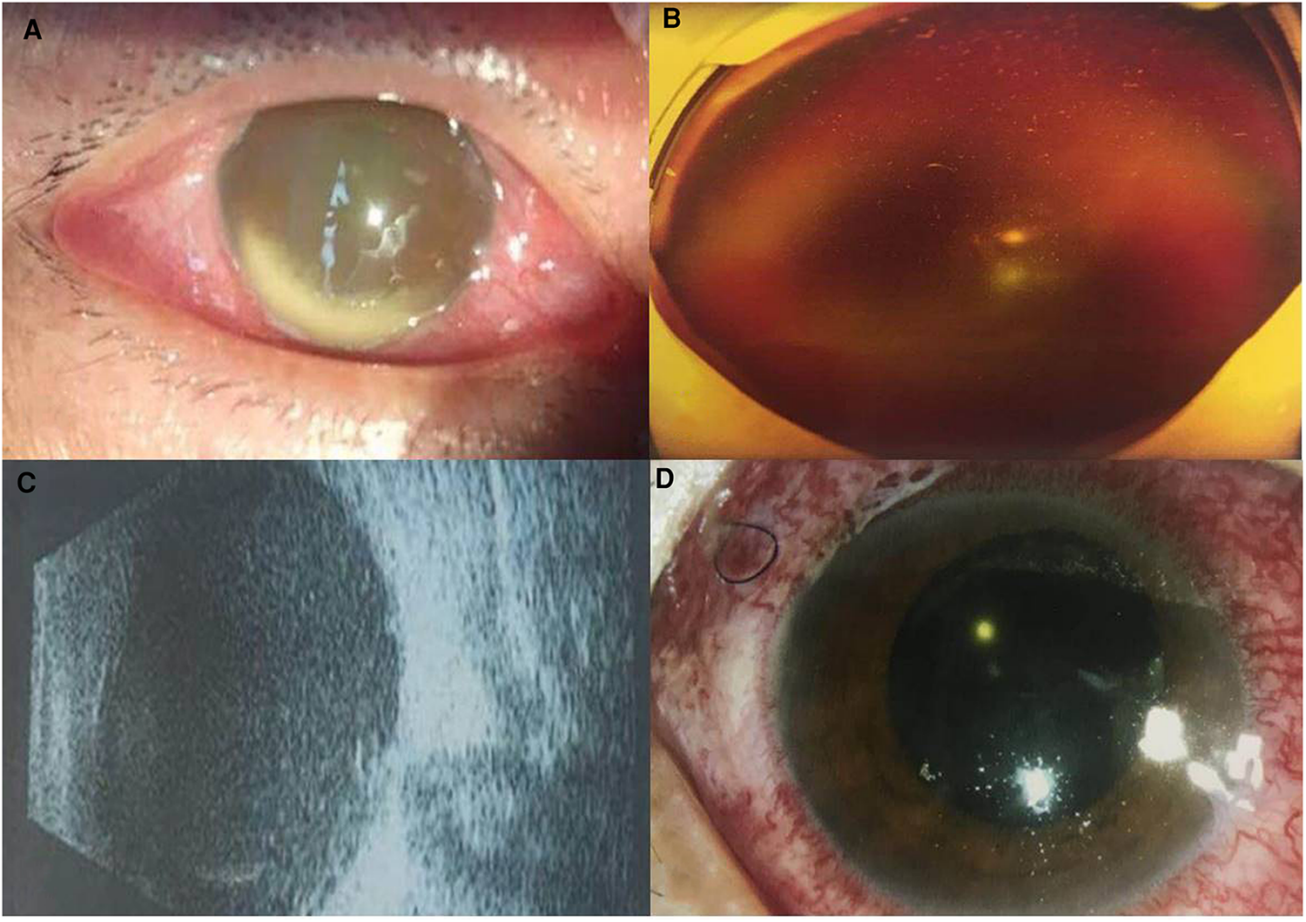
Ocular examination findings before and after surgery: case 1. (**A**) Hyphema accounting for 1/4 of the height of the anterior chamber at the bottom before surgery. (**B**) Vitreous hemorrhage before surgery. (**C**) B-ultrasound scan showed vitreous exudation with a small amount of hemorrhage after surgery (massive high-density shadows located under the vitreous). (**D**) Cornea and aqueous humour were clearer after surgery.

**Figure 2:**
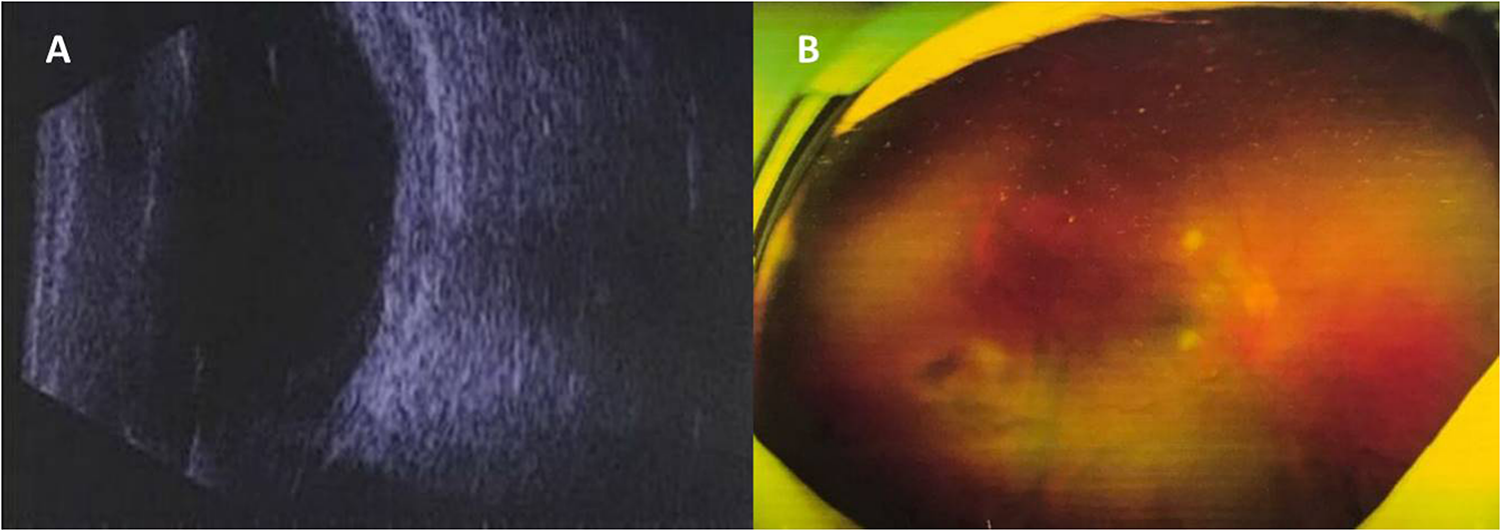
Fundus findings after surgery: case 1. (**A**) B-ultrasound scan showing slight turbidity of the vitreous body and vitreous organization after surgery. (**B**) Vitreous hemorrhage was partially absorbed and the optic nerve head could be seen faintly after surgery.

A 41-year-old male presented with complaints of pain and a foreign body (FB) that had entered his right eye. As stated, the patient had worked on hitting the fence with a hammer and was suddenly attacked by metallic stones from the flour fence below that entered his right eye. No history of unconsciousness occurred following the injury. He presented with no history of any other illness or injury previously. He had come to our hospital (Shenyang Fourth People's Hospital) about 3 h after the injury occurred since May 13, 2021. On examination, his best corrected visual acuity (BCVA) remained light perception, with a full-thickness cornea-scleral laceration wound without uveal tissue prolapse at 7 O'clock; the wound extended 2.5 mm backward from the limbus. Ophthalmic examination revealed some exudates in the anterior chamber within the affected eye. An obvious intraocular foreign body was shown by a B-ultrasound scan and was localized in the vitreous cavity without any damage to the retina and lens. The four stones had come into the vitreous cavity through the full-thickness cornea-scleral laceration wound and were located in the peripheral retina. Extraocular muscle movement was normal. Urgently, he received an IVI of 1 mg/0.1 ml vancomycin on the day after the ocular trauma. The left eye was within the normal limit. During preparation for further treatments, it was found that endophthalmitis occurred approximately 6 h after trauma. Anterior segment examination showed hyphema accounting for 1/4 of the height at the bottom of the shallow anterior chamber (Figure 1A). Some ocular symptoms such as conjunctival congestion, corneal edema, and aqueous humor turbidity also existed. The pupil of the eye dilated in response to injury, and the light reflection disappeared. Posterior segments of the eye manifested as vitreous hemorrhage, and the fundus was unclear (Figure 1B). It was expected from the B-ultrasound scan examination, which revealed a small amount of hemorrhage and no serious morphological damage surrounding the foreign bodies such as retinal detachment (Figure 1C). Immediately, four small stones were extracted from the only corresponding pars plana incision using a magnet without pars plana vitrectomy. A 20 g intravitreal foreign body magnet (Synergetics Inc., USA) was introduced into the vitreous body, and foreign bodies were gently dislodged and extracted via the wound. There was no impact on the retina and uvea. The intraocular fluid was also obtained, and its biochemical testing results indicated that the pathogenic microorganism was *Staphylococcus aureus*. On the second day, endophthalmitis was still serious and a traumatic cataract occurred. Hereinafter, he received IVI of 1.25% PI followed by a complete pars plana vitrectomy combined with expanding gas tamponade combined with irrigation of 0.025% PI, which was combined with cataract surgery with intraocular lens implantation. About 0.1 ml of 5% PI, which is an undiluted solution of PI, was taken in a 1 ml syringe and then 0.3 ml of saline solution was added. Three incisions in the flat part of the ciliary body were made by a 25G puncture knife. During the operation, the doctor found a little white purulent cluster in the vitreous body and on the retinal surface. After clearing the purulent clusters and vitreous body, the optic disc of the right eye was clear and pale red, the retina was flat, and the dark area of the macula was visible. Postoperative administration included intravenous cefoperazone + sulbactam (1107.4 mg + 1107.4 mg) (Zhuhai Jiayi Biotechnology Co., Ltd.) 2 × 2 g treatment twice daily for 1 week and topical 0.5% levofloxacin and 1% prednisolone acetate eye drop four times daily for 2 weeks. The BCVA of the involved eye stabilized at light perception 3 days after the surgery. The anterior segment showed that the hyphema had disappeared. The cornea and aqueous humour were clearer than before surgery. The pupil was dilated, avoiding synechia (Figure 1D). Furthermore, the B-ultrasound examination showed slight turbidity of the vitreous body and vitreous organization (Figure 2A). Ten days after the surgery, posterior segment examinations revealed that the vitreous hemorrhage was partially absorbed, and the optic nerve head could be seen faintly (Figure 2B). After 1 month, the BCVA increased to 0.7 (refer to decimal visual acuity of 1.0). Anterior segments of the eye condition recovered without conjunctival congestion. Moreover, the B-ultrasound examination revealed thickening of the ocular wall and formation of organizing strips connected with the retina. Six months later, the eye was quiet, and no obvious tears, holes, or detachment was seen in the retina. The patient’s vision remained 0.7 and was happy. The visual acuity was improved, maybe due to cornea edema, hyphema, and vitreous opacity being clear.

## Case 2

**Figure 3:**
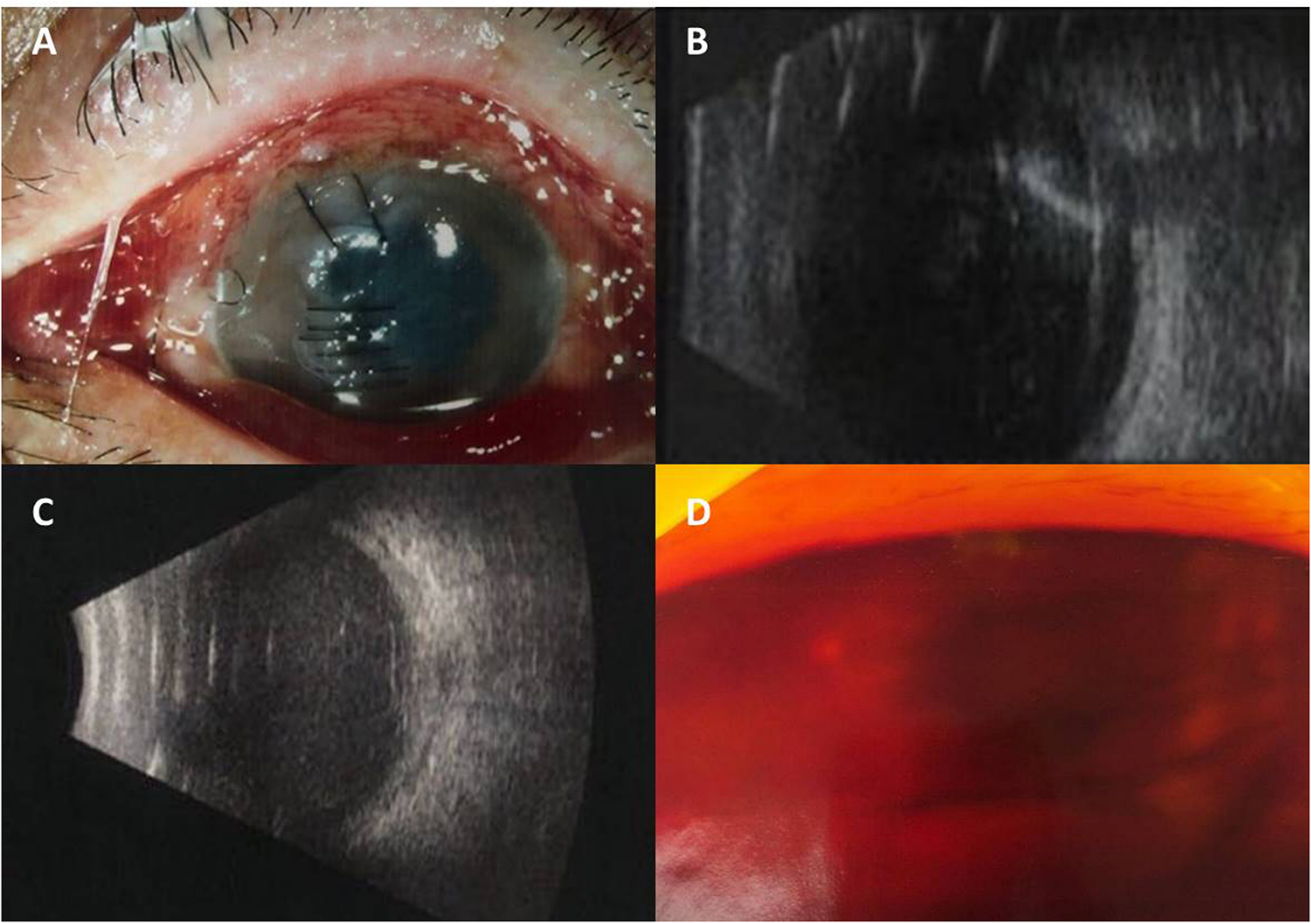
Anterior segmental findings before and after surgery: case 2. (**A**) At presentation, corneal laceration and edema were seen. (**B**) Vitreous organization and the echo of the foreign body before surgery. (**C**) Vitreous hemorrhage, edema of choroid and Tenon's capsule, and incomplete retinal detachment after surgery. (**D**) Vitreous hemorrhage slightly absorbed after surgery.

A 58-year-old male was referred to our hospital with complaints of his left eye being crushed by a stone accidentally during mowing grass on June 10, 2021. He had come to our hospital (Shenyang Fourth People's Hospital) about 1.5 h after the injury had occurred. His left eye's BCVA remained light perception with normal intraocular pressure. A slit lamp examination revealed that the traumatic type was a corneal laceration. Corneal edema was also present (Figure 3A), and other ocular parts were difficult to observe. The B-ultrasound scan showed a severe vitreous opacity without any posterior vitreous detachment, but the echo of the foreign body was observed (Figure 3B). Herein, a diagnosis of endophthalmitis was presumed, and vitreous samples were immediately obtained for pathogenic identification. After 2 days, the culture result indicated *Escherichia coli* infection, and he accepted an IVI of 1.25% PI followed by a complete pars plana vitrectomy combined with expanding gas tamponade with irrigation of 0.025% PI. Massive white purulent clusters in the vitreous body and on the retinal surface but without any retinal break in the retina were found during the surgery. Similar to that for case 1, 0.1 ml of 5% PI, which is an undiluted solution of PI, was taken in a 1 ml syringe and then 0.3 ml of saline solution was added. The scleral incision was 3.5 mm from the limbus. One of the sclerotomy sites was enlarged like the T or L letter to remove the foreign body. With the 20-gauge forceps, the foreign body was removed through an incision. It was a metal foreign body of 2 mm and 3 mm in length and less than 1 mm in diameter. There was no macular damage or circulatory disturbance on retinal observation during the surgery. Postoperative administration included intravenous cefoperazone + sulbactam (1107.4 mg + 1107.4 mg) (Zhuhai Jiayi Biotechnology Co., Ltd.) 2 × 2 g treatment twice daily for 1 week and topical 0.5% levofloxacin and 1% prednisolone acetate eye drop four times daily for 2 weeks. On the second day, the BCVA was still light perception. The B-ultrasound scan showed vitreous hemorrhage, edema of the choroid and Tenon's capsule, and incomplete retinal detachment (Figure 3C). Seven days after surgery, ophthalmoscopy showed that hemorrhage was absorbed (Figure 3D) and his BCVA recovered to 0.02. Six months later, the retina remained attached with slight fibrous proliferation and his vision improved to 0.1. The visual acuity impairments of this case might be due to damage to the retina and corneal laceration.

## Discussion

Traumatic eye injuries can attract microorganism and cause endophthalmitis, especially in the penetrating wound with intraocular foreign bodies. In this study, we used sealed PI (5% iodine solution: Shenyang GuangCai Medical Development Company, Shenyang). In these two cases, 0.025% PI-BSS PLUS was used to irrigate the anterior chamber at the beginning of vitrectomy for 10 min. The 0.025% PI solution was also used to irrigate the vitreous body for 10 min, and the irrigation solution was changed to the normal BSS solution before closing the surgery. There was a gap of more than 3 h between the IVI of 1.25% PI and vitrectomy.

In current cases, even after eliminating the pernicious influence of sterilizers, the physical removal of bacteria is also essential to reduce inflammatory reactions. In addition, the bacterial growth kinetics revealed that *Enterococcus faecalis* propagates rapidly in only 7 h (13) and *Candida albicans* propagates in 24 h (14). Considering these kinetics, initial treatment for traumatic endophthalmitis with ocular FB is the key step in preventing bacterial growth. In terms of the initial treatment for endophthalmitis, IVI of vancomycin or ceftazidime has been widely recommended (15, 16); however, the production of multidrug-resistant organisms that are resistant to both these antibiotics has already been reported. Moreover, vancomycin requires 8 h to exert its bactericidal action, which may miss the best opportunity to salvage the remaining vision (17). Notably, adjusting the concentration of antibiotics for IVI is also rather complicated because the ocular tissue is delicate and may be damaged by corrosive substances. IVI of antibiotics might be effective for maintaining a postoperative intraocular environment free from bacteria. However, postoperative hemorrhagic occlusive retinal vasculitis associated with intracameral vancomycin as prophylaxis during cataract surgery has been reported (18). Thus, we must be cautious when injecting antibiotics directly into the eyes. PI for intraocular tissues and its potential toxicity have been studied in detail (19), which showed that 0.025% PI was testified to be safe for the corneal endothelial cells, anterior chamber, and vitreous body in rabbit eyes; thus, the introduction of PI for treating endophthalmitis is meaningful to some extent. In the current two cases, we used expanding gas to prevent retinal detachment from pars plana or pars plicata and to prevent further bleeding in case 1. Furthermore, we used expanding gas to prevent retinal detachment and further bleeding in case 2 because massive white purulent clusters on the retinal surface were suggestive of retinal fragility even without breaks.

Previously, Kim et al. also reported that repeated irrigation of the operative field with 0.25% PI is safe for ocular tissues and highly bactericidal in a wide range of ocular surgeries (20). They revealed that the half-life period of PI was approximately 3 h in the vitreous body. In addition, electroretinography (ERG) and histologic examinations of the retina were performed to verify that both 0.1% and 0.3% PI were tolerable. Furthermore, 0.025% PI-balanced salt solution (BSS) PLUS (Alcon Laboratories, Fort Worth, TX) is a bactericidal drug when used as an irrigation solution for vitrectomy in eyes with endophthalmitis (21). IVI of 1.25% PI followed by vitrectomy using 0.025% PI irrigation for treating endophthalmitis was proved to be effective (22). Above all, this therapy is anticipated to become a new treatment for endophthalmitis caused by multidrug-resistant organisms, such as vancomycin-resistant bacteria and fungal organisms.

However, there were some limitations of the current report because we still lack a large number of clinical cases treated with this therapy, which may be more convictive. Further investigation of this therapy is still needed. Similarly, Nakasazuka et al. have shown good clinical outcomes in postoperative endophthalmitis treated with intravitreal 1.25% PI injection followed by vitrectomy using 0.025% PI in the irrigation solution (22). However, there are limited reports on the safety and efficacy of dilute PI as an alternative or adjunct to intravitreal antibiotics. It may be a good alternative in resource-poor communities or cases of multidrug-resistant infections.

In conclusion, we postulated that IVI of 1.25% PI followed by vitrectomy using 0.025% PI irrigation for treating endophthalmitis with traumatic foreign bodies could be considered an optimal initial treatment for traumatic endophthalmitis and may be useful from the viewpoint of maintaining the disease.

## Data Availability

The original contributions presented in the study are included in the article/Supplementary Material; further inquiries can be directed to the corresponding author/s.
